# The Medical Officer

**Published:** 1923-09

**Authors:** 


### THE MEDICAL OFFICER OF HEALTH.

### HIS PROFESSIONAL EDUCATION AND TRAINING.

NOWADAYS the young medical graduate, on leaving his medical school, has some knowledge of the preventive aspects of Medicine. This change in the teaching of medical subjects has been gradually brought about during recent years, and now the resolutions of the General Medical Council in regard to Professional Education adopted in May, 1922, and operative since January 1, 1923, give a definite preventive bias to medical study. The first and most important of these resolutions is that throughout the whole period of study the attention of the student should be directed by the teachers to the importance of the preventive aspects of Medicine. Considering the large amount of public interest that is now taken in preventive medicine and the vast field that lies open to the medical man who is anxious to find out means by which the health of the individual or of the community may be maintained, it is not unlikely that many young graduates will be attracted in the future to the study of communal hygiene and the acquisition of a degree or diploma in State Medicine or Public Health.

### The New Rules.

At the same time that the General Medical Council were engaged in the alteration of the medical curriculum, they had under consideration alterations in the Public Health curriculum. These alterations come into force on January 1, 1924. The new rules are as follows:—

*Rule* 1.—A period of not less than two years shall elapse between the attainment by a candidate of a registrable qualification in Medicine, Surgery and Midwifery, and his admission to the Final Examination for a Diploma or Degree in Sanitary Science, Public Health or State Medicine.

*Rule* 2.—The curriculum for a Degree or Diploma in Sanitary Science, Public Health or State Medicine shall extend over a period of not less than twelve calendar months subsequent to the attainment of a registrable qualification.

*Rule* 3.—Every candidate shall produce evidence of having attended, during a period of not less than five months, at an institution approved by the Licensing Body granting the Diploma or Degree, practical instruction in:—Bacteriology and Parasitology (including Medical Entomology), especially in their relation to diseases of man, and to those diseases of the lower animals which are transmissible to man (at least 180 hours, of which 150 must be in laboratory work); Chemistry and Physics in relation to Public Health (at least ninety hours, seventy in laboratory); Meteorology and Climatology in relation to Public Health (at least ten hours).

*Rule* 4.—Every candidate shall produce evidence of having received, during not less than eighty hours, at an institution or from teachers approved by the Licensing Body granting the Diploma or Degree, instruction in the following subjects:—The Principles of Public Health and Sanitation (thirty hours); Epidemiology and Vital Statistics (twenty hours); Sanitary Law and Administration (including Public Medical Services) (twenty hours); Sanitary Construction and Planning (ten hours).

*Rule* 5.—Every candidate shall produce evidence that he has attended for three months on the clinical practice of a recognised Hospital for Infectious Diseases, and has received therein instruction in the methods of administration. At least thirty daily attendances of not less than two hours in each week shall be required.

*Rule* 6.—Every candidate shall produce evidence that he has during a period of not less than six months been engaged in acquiring a practical knowledge of the duties, routine and special, of Public Health Administration under the supervision of a Medical Officer of Health, who shall certify that the candidate has received, from this Officer or other competent Medical Officer, during not less than three hours on each of sixty working days, practical instruction in these duties, and also those relating to:—Maternity and Child Welfare Service; Health Service for Children of School Age; Venereal Diseases Service; Tuberculosis Service; Industrial Hygiene; Inspection and Control of Food, including meat and milk. Certificates of having received the prescribed instruction in Public Health Administration must be given by a Medical Officer of Health who devotes his whole time to Public Health work; or by the Medical Officer of Health of a Sanitary Area having a population of not less than 50,000, or in Ireland the Medical Superintendent Officer of Health of a County or County Borough having a population of not less than 50,000.

### Public Health Work as a Career.

Thus the new Public Health Regulations require that a period of not less than two years shall elapse between the attainment by a candidate of a registrable qualification in Medicine, Surgery and Midwifery, and his admission to the Final Examination for a Diploma or Degree in Sanitary Science, Public Health or State Medicine. The General Medical Council avowedly state that the purpose of this Regulation is to provide opportunity for candidates for the Diploma or Degree in Sanitary Science, Public Health or State Medicine, to pass from the state of pupilage to that of responsible practitioners, to give mature consideration to the obligations and duties involved in the work of the Public Health Service, and to acquire direct experience of medical work in a responsible capacity either in practice or in hospital or laboratory appointments. The Council believe that, in the public interest, the time has arrived to adapt the Diploma to candidates who are seriously intending to take up Public Health work as a career.

### The Value of Hospital Appointments.

During this period of two years the Public Health Undergraduate should, in the first instance, increase his knowledge of clinical work, preferably in hospital appointments. The value of these appointments is that generally they enable the medical man to keep close and continuous observation on his cases, and properly co-relate the symptoms and signs of disease with pathological and bacteriological findings and therapeutics. He is able, in fact, to make a complete study of his cases which is not always possible in general practice. Having equipped himself as a good clinician and having been trained in some of the aspects of Preventive Medicine in the general Medical Curriculum, he is in a position to study with advantage the special curriculum laid down by the General Medical Council for Diplomas or Degrees in Sanitary Science, Public Health or State Medicine.

### Getting a Practical Insight.

The course arranged under the Rules seeks to give the student a practical insight into Public Health work as practised to-day. If he takes full advantage of the facilities afforded, he should enter the Public Health profession with a clear conception of general sanitation and communal hygiene and a working knowledge of the various Services which have been put into operation by Local Authorities for dealing with Maternity and Child conditions, Tuberculosis, Venereal Diseases and the like.

### Co-operation with Local Authorities.

In relation to the training of Medical Officers of Health it is necessary to interest local authorities and to stimulate them to provide facilities for training. After all, it is the local authorities who will employ Medical Officers of Health, and it is desirable therefore that, even during training, there should be some relationship between the Public Health undergraduate and his future employer. Such a relationship established during the years of training would probably enable the work of the Medical Officer of Health later to be the more easily undertaken and accomplished.

### A Strenuous Training.

The professional education and training of a Medical Officer of Health means the expenditure of a considerable sum of money and a period of preparation of not less than seven years. It is doubtful, having regard to the emoluments which local authorities offer, if this long period of training and education is justifiable. We know that it is necessary in order to equip men with the necessary knowledge for the work which the local authorities have to do but apparently are not prepared to pay for. At the Portsmouth meeting of the British Medical Association a better scale of salaries was adopted for Medical Officers of local authorities, and although we do not consider these by any means more than adequate, we hope that they will be the means of attracting promising graduates to a service which is of the utmost importance to the National Health.

